# Ethnopharmacological Survey and Antisickling Activity of Plants Used in the Management of Sickle Cell Disease in Kikwit City, DR Congo

**DOI:** 10.1155/2020/1346493

**Published:** 2020-10-26

**Authors:** Jules Munganga Kitadi, Prince Pambi Mazasa, Damien Sha-Tshibey Tshibangu, Félicien Mushagalusa Kasali, Dorothée Dinangayi Tshilanda, Koto-Te-Nyiwa Ngbolua, Pius Tshimankinda Mpiana

**Affiliations:** ^1^University of Kikwit, Kwilu, Kikwit, Democratic Republic of the Congo; ^2^Department of Biology, Faculty of Science, University of Kinshasa, P.O. Box 190, Kinshasa, Democratic Republic of the Congo; ^3^Department of Chemistry, Faculty of Science, University of Kinshasa, P.O. Box 190, Kinshasa, Democratic Republic of the Congo; ^4^Department of Pharmacy, Faculty of Pharmaceutical Sciences and Public Health, Official University of Bukavu, P.O. Box. 570, Bukavu, Democratic Republic of the Congo

## Abstract

**Background:**

Sickle cell disease or drepanocytosis is the most known hemoglobin abnormality in the world. Recently, many medicinal plants used in the management of sickle cell disease in African traditional medicine have shown *in vitro* antisickling activity.

**Objective:**

This research study aims to document some Congolese plant species used in the management of sickle cell disease in Kikwit city and investigate their pharmacological property *in vitro*.

**Methods:**

A cross-sectional survey was done from June 2015 to March 2016 among 26 traditional healers in Kikwit city, Democratic Republic of Congo. Emmel test was used to assess *in vitro* antisickling activity. Habitat, morphological, biological types, phytogeographical distribution, local names, and used parts of these plant species were also determined.

**Results:**

Obtained results show that 23 plant species belonging to 16 families are used. The leaves represent the most used part (70%). Antisickling investigation showed that aqueous extracts of 18 plants (78%) exhibit a change in the shape of a sickle cell into a normal one with a normalized rate of at least 70%, confirming the *in vivo* effect observed by traditional healers when used as herbal medicine traditionally in the management of sickle cell disease. The evaluation of perimeter, surface, and radius of untreated and treated sickle red blood cells showed a significant difference (*p* < 0.05) as modification indicators of the red blood cell shape. *Alchornea cordifolia, Alternanthera bettzickiana, Annona senegalensis, Dissotis brazzae, Hypoxis angustifolia*, and *Justicia secunda* presented a very high antisickling activity with normalization >70%. Otherwise, *Dissotis brazzae* was the most active plant with a minimal concentration of normalization (MCN) of 11 *μ*g/mL and a maximal normalization rate (NRmax) of 86%.

**Conclusion:**

Almost 80% of the plants studied showed interesting antisickling activity and justified their use in traditional medicine. The isolation of the molecules responsible for the biological activity of the most active plants and the determination of their chemical structures is under investigation.

## 1. Introduction

Sickle cell disease (SCD), also called sickle cell anemia or drepanocytosis, is one of the most common hemoglobin abnormalities in the world [1–5]. About 50 million people are affected in the world, mainly sub-Saharan Africans and their immigrants, especially to America. Nevertheless, this hemoglobin disease is also found in some parts of India and Saudi Arabia [6–8]. In certain African regions, carriers of S hemoglobin can reach 20% of the population with a prevalence of 25–30% in Central Africa, including the Democratic Republic of the Congo (RDC). Indeed, in the DRC, about a million and a half people are affected by this genetic disease [9–12].

SCD is a painful disease due to a genetic mutation conducting to the substitution of a polar amino acid (glutamic acid) by a nonpolar one (valine) on the sixth position of the beta-globin chain [13]. This change decreases the affinity of hemoglobin for oxygen. Furthermore, this structural modification influences the solubility of hemoglobin conducting abnormal hemoglobin, hemoglobin S (HbS), to polymerize inside the erythrocytes into a gel or further into fibers, causing the change of the red blood cell (RBC) shape from the normal biconcave form into the sickle form in hypoxic conditions. The HbS polymerization and the sickling of the RBC are responsible for sickle cell disease to basic pathological processes, the painful vaso-occlusive crises, and chronic hemolytic anemia. The occlusion of microvessels' circulation leads to chronic tissue ischemia and infarction affecting the bone, lungs, brain, kidneys, or spleen that conduct to complications in sickle cell disease [13–15].

Many therapeutic approaches are proposed to relieve sickle cell disease patients from this painful and chronic disease. These include the medullary transplantation, repeated blood transfusion, and the use of hydroxyurea. These approaches are unfortunately ineffective, expensive for the African population, or present high risk of transmission of other infections [13, 15, 16]. Therefore, there is a need for more affordable and effective treatments for this disease. Medicinal plants and traditional medicine seem to offer an adapted solution to African populations. In fact, according to the World Health Organization (WHO), 80% of the world's population, mainly in Africa, use medicinal plants for some aspects of primary health care [17, 18]. The DRC is by far the African country with the highest biodiversity with richest flora in the world. Surveys conducted by our research team around big cities revealed more than 120 medicinal plants used in traditional medicine in the management of SCD [4–6, 14, 19–29].

The present study aims to document and investigate *in vitro* the medicinal plants used in Congolese traditional medicine in the management of SCD in Kikwit city.

## 2. Materials and Methods

### 2.1. Study Area

**Figure 1:**
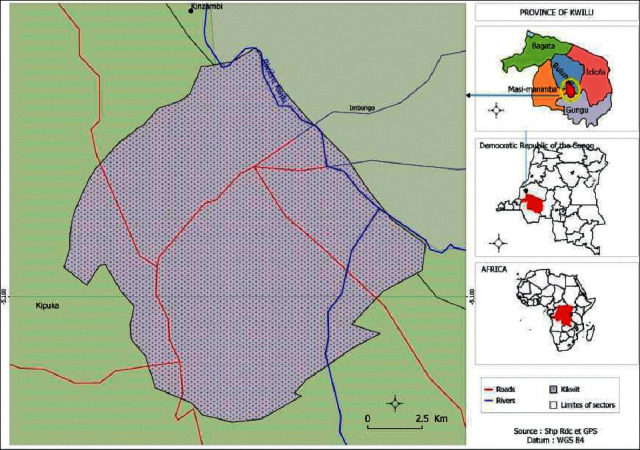
Kikwit city in the Democratic Republic of the Congo.

The survey was conducted in Kikwit city, province of Kwilu, located in the southwest part of the Democratic Republic of the Congo at 500 km west of the capital city Kinshasa (Figure 1). Kikwit's population was estimated to be 1,000,000 inhabitants in a total area of 92 km [19].

### 2.2. Ethnopharmacological Survey

The ethnopharmacological survey was carried out from June 2015 to March 2016. Twenty-six traditional healers from different ethnic groups were identified and questioned on a voluntary basis following the principles laid out in the Declaration of Helsinki with minor modification [20]. The data checklist was divided into three sections:

  Personal information such as name, age, sex, marital status, ethnic group of the informant, and study level
  Plant data (scientific and vernacular names, village survey, parts used, and the method of harvesting stage or degree and organ development)
  Plant therapy data: methods of preparation and administration (transaction and pharmaceutical form, concentration of the organ dose, frequency of taking, and instructions)

The identification of collected plants was carried over by comparison with vouchers referenced at the herbarium of Faculty of Sciences, University of Kinshasa. Voucher specimens are on deposit at the same herbarium.

### 2.3. Floristic Characterizations of Collected Plants

The collected medicinal plants are characterized by their biological, morphological, and habitat types and by their phytogeographical distribution [17, 21]. The following biological types were selected: mesophanerophytes (MsPh), microphanerophytes (McPh), nanophanerophytes (NPh), chamaephytes (Ch), therophytes (Th), therophytes scapose (Tsc), erected chamaephytes (Ch er), climbing chamaephytes (Ch cl), and geophytes (G).

The morphological types inventoried are tree (T), shrub (Sh), subshrub (Ssh), annual herb (Ah), vivace herb (Vh), perennial herb (Ph), and liana (L).

Habitat types are distributed as follows: cultured (Cult), subspontaneous cultured (Cult ssp), forest (Fo), fallow (Fal), and ruderal (Rud).

The phytogeographical types of distribution are Afro-tropical (AT), Bas-Guineo-Congolese (BGC), Guineo-Congolese (GC), Guineo-Congolese-Zambezean (GCZ), and pantropical (Pan).

### 2.4. Evaluation of Use Value and Informant Consensus Factor Value

Use value (UV) that evaluates the relative importance of each medicinal species based on its relative use among informants was calculated using the following formula:

(1) $$\begin{array}{c} \text{UV}=\frac{U_{i}}{N_{i}}, \end{array}$$

where *U*_*i*_ is the number of use reports cited by each informant for a given plant species when *N*_*i*_ is the total number of informants interviewed for a given plant species.

The usage value agreement is defined as the relative importance of each plant type known to be used as herbal medicine. This index is useful in identifying plants with the highest use (most frequently mentioned) in the treatment of a disease with a given informant consensus factor (ICF) value. ICF (ranging from 0 to 1) is used to deduce the homogeneity in the data on the use of a specific plant to treat a certain disease. The informant consensus factor value was calculated using the following formula:

(2) $$\begin{array}{c} \text{ICF}=N_{c}-\frac{N_{\text{ps}}}{N_{c}-1}, \end{array}$$

where *N*_*c*_ is the number of plant use reports (citation number) per each category and *N*_ps_ is the number of plant species (taxa used). A high value of ICF (close to 1) shows that a reduced number of plant species are quoted by a large number of informants for a specific type of treatment, indicating the consistence of the use of this medicine [22].

### 2.5. Biological Samples

Blood samples used for antisickling evaluation were collected from known sickle cell disease patients treated in Centre de Médecine Mixte et d'Anémie *SS* in Kinshasa, DRC. None of the patients had been transfused recently with Hb AA blood. All antisickling experiments were conducted with freshly collected blood. In order to confirm their *SS* nature, the aforementioned blood samples were first characterized by hemoglobin electrophoresis on cellulose acetate gel, as previously reported [7, 8, 14]. They were found to be *SS* blood and were then stored at ±4°C in a refrigerator. Informed consent was obtained from all the patients selected in the study. Ethical clearance for the use of sickle blood cells was strictly observed according to international rules.

### 2.6. Antisickling Assay

Antisickling assay was carried out using Emmel test as previously reported [6, 9, 18]. In brief, sickle cell blood was diluted with 150 mM phosphate buffer saline (NaH_2_PO_4_ 30 mM, Na_2_HPO_4_ 120 mM, and NaCl 150 mM) and mixed with an equivalent volume of 2% sodium metabisulfite. A drop of the mixture was spotted on a microscope slide in the presence of aqueous plant extracts and covered with a cover slip. Physiologic solution was used as the negative control when parahydroxybenzoic acid was used as the positive control. Paraffin was applied to seal the edges of the cover slip completely to create hypoxia condition.

### 2.7. Data Analysis

Different parameters have been screened in red blood cells, including the area, perimeter, and radius of each RBC using a computer-assisted image analysis system (Motic Images 2000, version 1.3; Motic China Group Co., Ltd), and statistical data analysis was processed using Microcal Origin 8.5 package software.

## 3. Results and Discussion

### 3.1. Ethnobotanical Analysis

Table 1 summarizes families, species, vernacular names, used parts, and use values of plants used in the management of sickle cell disease in Congolese traditional medicine in Kikwit city.

**Figure 2:**
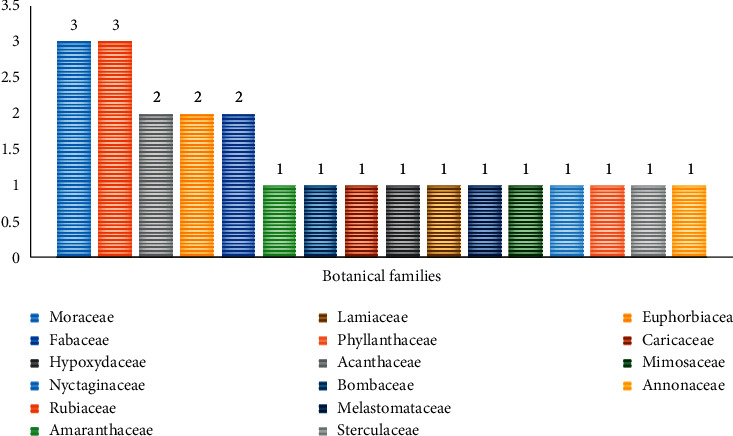
Distribution of species by family.

**Figure 3:**
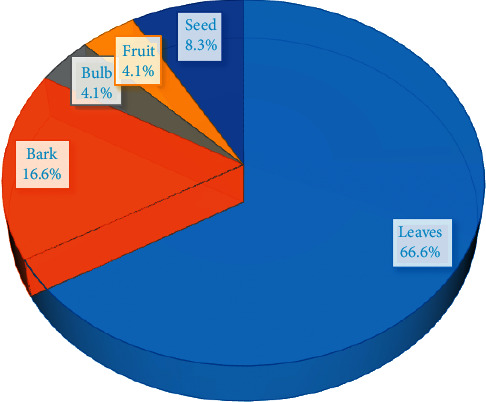
Percentage of used parts of plants.

23 plant species from 16 botanical families cited in different local names, especially Kikongo (sometimes in Lingala and rarely in French), are used in the management of sickle cell disease in Kikwit city. Figures 2 and 3 give the number of species by family used parts, respectively.

According to the results (Figure 2), Rubiaceae and Moraceae families have been represented by three species. However, three families including Euphorbiaceae, Acanthaceae, and Fabaceae had two species, each one and the remaining possessing only one plant species by family.

Figure 3 shows that leaves represent the most used part (66.6%) when barks represent 16.6%. The use of leaves could be justified by the abundance of chemical groups they contain. In fact, leaves are known as the main synthesis site of secondary metabolites in plants and are the most commonly used plant parts by traditional medicinal practitioners [17, 21].

### 3.2. Use Value

Eight plants (*Justicia secunda*, *Vigna unguiculata*, *Alternanthera bettzickiana*, *Alternanthera bettzickiana*, *Carica papaya*, *Annona senegalensis*, *Alchornea cordifolia*, and *Hypoxis angustifolia*) have presented a use value higher than 0.6. They are the most cited plants, and therefore, they are the most used plants in the management of sickle cell disease in Kikwit. According to previous antisickling studies, all cited plant species, except *Alternanthera bettzickiana* and *Hypoxis angustifolia*, are well known [4–6, 14, 23–33].

A*lternanthera bettzickiana* repens (L.) Link displayed both high UV and a large consensus of use among traditional healers indicating the consistence of the use of this plant species as antisickling medicine.

### 3.3. Ecological Status of Plant Species

Ecological characteristics of these plants are given in Table 2.

### 3.4. Habitat Types

**Figure 4:**
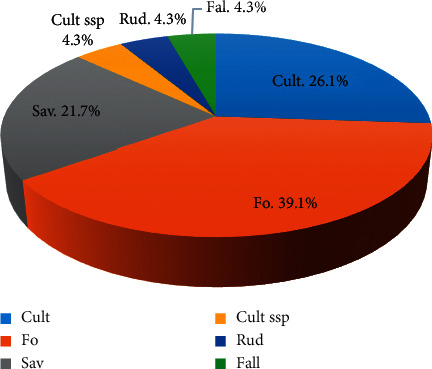
Habitat types of plant species.

Types of habitat are presented in Figure 4. This figure shows that the main habitat type of plants used in the management of sickle cell by traditional healers in Kikwit is forest (39.1%) followed, respectively, by cultured (26.1%) and savanna (21.7%). Fallow, ruderal, and subspontaneous cultured represent each one 4.3%.

### 3.5. Morphological Types

**Figure 5:**
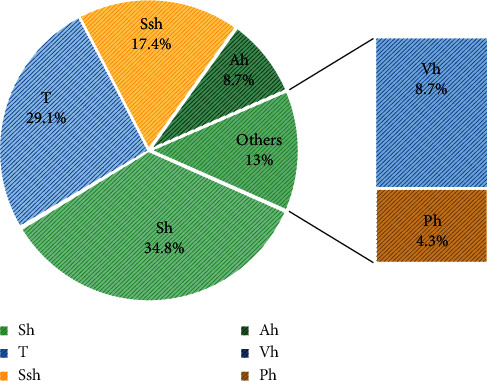
Morphological types of plants species.

Figure 5 represents morphological types of used plants. Shrub represents the most morphological types (34.8%) followed by tree (26.1%), subshrub (17.4%), and annual herb (8.7%). Vivace herb and perennial herb both represent 13%. Furthermore, 14 plant species, up to 23 representing 61% of species, are trees or shrubs. Only five plant species (21%) are herbs. Thus, a correct use of leaves of these plants can permit a sustainable exploitation of these species in traditional medicine.

### 3.6. Biological Types

**Figure 6:**
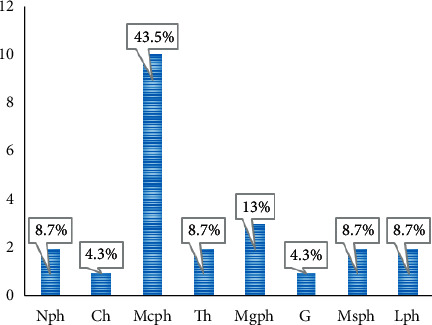
Biological types of plant species.

Biological types are listed in Table 2 and presented in Figure 6.

### 3.7. Phytogeographical Distribution

**Figure 7:**
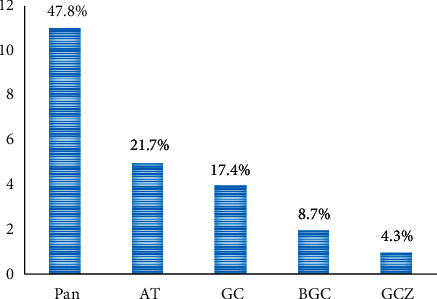
Phytogeographical distribution of plants.

Phytogeographical distribution of plants species is given in Figure 7.

Microphanerophyte is the main biological type and represents 43.5% of identified plant species. Pantropical represents the most phytogeographical distribution (47.8%).

### 3.8. *In Vitro* Antisickling Activity

**Figure 8:**
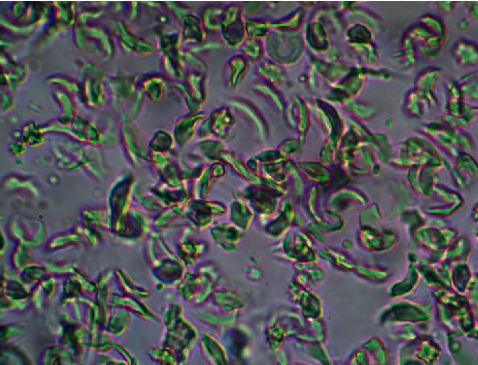
Erythrocytes' morphology of untreated sickle cell blood (only saline solution, negative control) (x500) (NaCl 0.9%; Na_2_S_2_O_4_ 2%).

**Figure 9:**
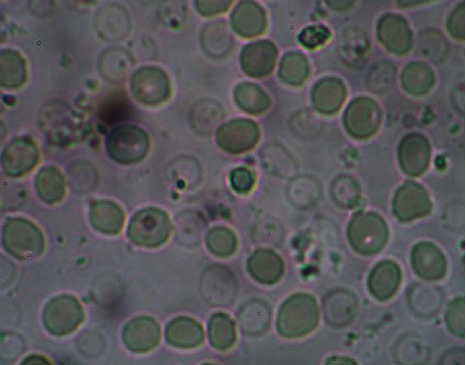
Erythrocytes' morphology of sickle cell blood in the presence of parahydroxybenzoic acid (0.8 *μ*g/mL) (x500) (NaCl 0.9%; Na_2_S_2_O_4_ 2%).

**Figure 10:**
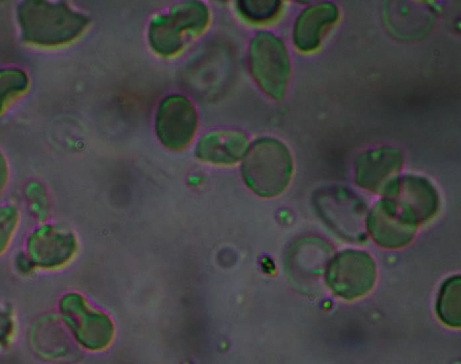
Erythrocytes' morphology of sickle cell blood in the presence of the aqueous leaves' extract of *Dissotis brazzae* (20 *μ*g/mL) (x500) (NaCl 0.9%; Na_2_S_2_O_4_ 2%).

**Figure 11:**
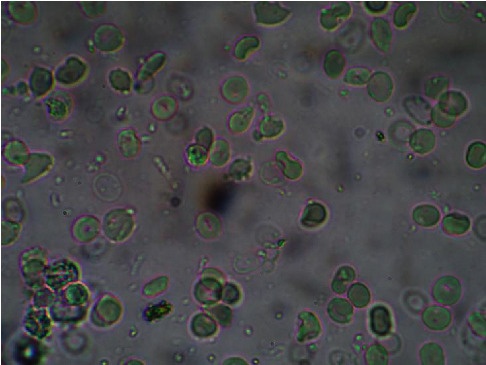
Erythrocytes' morphology of sickle cell blood in the presence of the aqueous leaves' extract of *Alternanthera bettzickiana* leaves (20 *μ*g/mL) (x500) (NaCl 0.9%; Na_2_S_2_O_4_ 2%).

The results of antisickling evaluation *in vitro* are represented in Figures 8–11. Figures 8 and 9 represent microscopic images of negative and positive controls when Figures 10 and 11 show morphologies of RBC treated with aqueous plant extracts, *Alternanthera bettzickiana*, and *Dissotis brazzae*, respectively.

Figure 8 indicates that the majority of red blood cells is sickled. It means and supports that collected blood is from the sickle cell disease patient.  Figure 9 indicates that all blood cells have a normal circular form due to the normalization effect of the positive control.

Figures 10 and 11 show that, in the presence of *D. brazzae* and *A. bettzickiana* aqueous extracts, red blood cells (RBCs) have a circular (biconcave) and normal shape indicating the antisickling effect of these extracts. This situation, *in vitro* activity, confirms the *in vivo* effect observed by traditional healers when using an aqueous decoction of these medicinal plants in the management of sickle cell disease in Kikwit city.

The same antisickling activity was found in the other 21 medicinal plants used in the management of sickle cell disease in Kikwit (Table 3).

Table 3 shows that 18 plants (78%) permit a change of shape of a sickle cell into a normal one with a normalized rate of at least 70%. This indicates that the information given by traditional healers is validated by *in vitro* tests. The same results were found in plants used in the management of sickle disease in other Congolese cities [4–6,14,23–33].

Six of these plants (*Alchornea cordifolia* Müll. Arg., *Annona senegalensis; Carica papaya; Hymenocardia acida; Justicia secunda;* and *Vigna unguiculata*) were already cited elsewhere by Congolese traditional healers in the management of sickle cell disease, and they have shown *in vitro* antisickling activities [4–6, 14, 23–33].

Table 4 gives use values of medicinal plants used in the management of sickle cell disease in Kikwit city.

*Hypoxis angustifolia* Lam., *Hymenocardia acida* Tul, and *Vigna unguiculata* (L.) Welp are among the most cited species in Kikwit.

In order to quantify the observed shape modification, perimeter, surface, and radius of untreated and treated sickle RBCs with plant extracts were evaluated.

Table 5 shows calculated sickle red blood cell perimeter, surface, and radius modification after treatment of *D. brazzae* and *A. bettzickiana* aqueous leaves' extracts.

Values (means ± S.D) in Table 5 show a significant difference (*p* < 0.05) between the negative control compared to values from the two extracts. This confirms the antisickling activity of these plant aqueous extracts. Furthermore, the values obtained from the two plant extracts are in the same range to those obtained in previous studies [4–6, 23–28, 30–33]. Used software cannot calculate the radius of untreated sickle red blood cells because the shape of cells was elongated, not circular. However, in the presence of plant extracts, cell shape becomes circular, and the radius could be calculated.

Indeed, hydrophobic interactions between hemoglobin S (HbS) molecules conduct to intracellular hydrophobic interactions that induce intracellular polymerization. This hemoglobin aggregation modifies the erythrocyte shape conducting to a sickled form. Plant extracts contain molecules that can interact with HbS molecules and compete with their polymerization [12, 32, 33]. This permits sickle red blood cells to return to their normal biconcave form.

**Figure 12:**
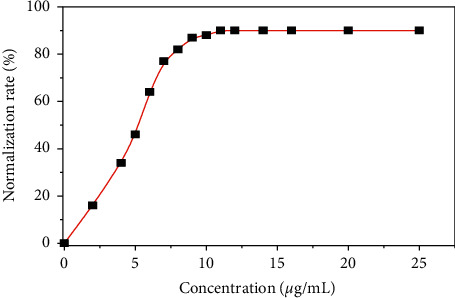
Evolution of the normalized rate of the sickle cell shape with the concentration of *D. brazzae* aqueous extracts.

**Figure 13:**
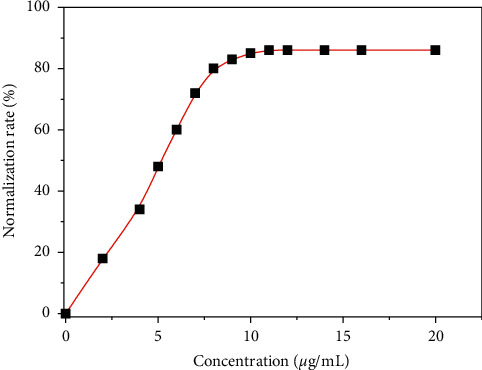
Evolution of the normalization rate of the sickle cell shape with the concentration of D. brazzae aqueous extracts.

The normalized rate can be calculated by following the percentage of erythrocytes that regain the normal shape in function of the concentration of plant extracts. Figures 12 and 13 give an illustration of the normalization of the aqueous extract of *D. brazzae* and *A. bettzickiana*, respectively.

These curves show that the percentage of the drepanocytes, which return to the normal shape, increases with the extract concentration indicating that the normalization rate is dose-dependent. The minimal concentration of normalization (MCN) obtained is 0.9 and 11.0 *μ*g/mL with a maximal normalization rate (NRmax) of 90 and 86% for *D. brazzae* and *A. bettzickiana*, respectively. This indicates that the aqueous extract of the two plants has very high NRmax with low MCN, but *D. brazzae* is a little more active than *A. bettzickiana*. The same behavior was found in other Congolese medicinal plants used in the management of sickle cell disease, but the obtained value of MCN and NRmax depends on the plant species [4, 5, 9, 23–33].

## 4. Conclusion

The traditional healers of Kikwit (DRC) use 23 plant species in the management of sickle cell disease. Eighteen plant species showed a high antisickling activity *in vitro* with a normalized rate of at least 70%. The present results validate and support the local uses of plants in the treatment of diseases, especially sickle cell disease. Most of these plants have already been cited for their antisickling activity in other parts of the DRC and in the literature. Two species including *Alternanthera bettzickiana* and *Dissotis brazzae* deserve in-depth investigations to meet scientific demands for clinical studies. Furthermore, it is crucial to isolate their bioactive compounds in order to understand their pharmacological mechanisms.

## Figures and Tables

**Table 1:** Plants used in the management of sickle cell disease in Kikwit.

Family	Plant species	Vernacular name	Used part	Use value	FCI
Acanthaceae	*Justicia secunda* Vahl.	Matiti ya ba témoins de Jéhovah (Lingala)	Leaves	3.58	0.58
	*Rungia grandis* T. Ander	—	Bark	3.34	0.55
Amaranthaceae	A*lternanthera bettzickiana* repens (L.) Link	Matiti ya ba témoins de Jéhovah (Lingala)	Leaves	5.12	0.68
Annonaceae	*Annona senegalensis* Pers.	Kilolo, nlolo (Kikongo)	Leaves	4.12	0.58
Bombaceae	*Ceiba pentandra* (L.) Gaertn.	Kapokier, fromager (French), mfuma (Lingala)	Leaves	4.00	0.63
Caricaceae	*Carica papaya* L.	Payipayi (Lingala)	Leaves, fruit	5.00	0.68
Euphorbiaceae	*Alchornea cordifolia* Müll. Arg.	Mbunji-mbunji (Lingala), kibunsi (Kikongo)	Leaves	4.56	0.7
	*Bridelia ferruginea* Benth.	Kimuindu ki nseke (Kikongo)	Leaves	3.67	0.72
Fabaceae	*Vigna unguiculata* (L.) Welp.	Mbwengi (Lingala, Kikongo)	Seeds	4.34	0.68
	*Millettia laurentii* De Wild	Koboto (Kikongo), ntoka (Kikongo), wenge (Lingala)	Leaves	4.22	0.62
Hypoxidaceae	*Hypoxis angustifolia* Lam.	Ditungulu ya mfinda (kikongo), litungulu (Lingala)	Bulbs	4.00	0.63
Lamiaceae	*Leocus africanus* (Bak. ex. Elliot) J. K. Morton	Zangazanga (Kikongo)	Bark	4.89	0.65
Melastomataceae	*Dissotis brazzae* Cogn.	Mampa ya mfinda (Kikongo)	Leaves	4.45	0.67
Mimosaceae	*Albizia ferruginea* (Guill. et Piero) Benth.	Sela (Kikongo)	Bark	4.22	0.68
Moraceae	*Treculia africana* Decne	Ngungi (Kikongo)	Leaves	3.78	0.58
	*Ficus asperifolia* müg	Kikuya, kuya (Kikongo)	Leaves	3.89	0.64
	*Ficus tremula* Warb.	—	Leaves	4.34	0.63
Nyctaginaceae	*Bougainvillea* sp comm. ex Juss	Bintuntu ya nzanguka (Kikongo)	Leaves	4.67	0.63
Phyllanthaceae	*Hymenocardia acida* Tul	Dikasu, kigete, ngete (Kikongo)	Leaves	4.67	0.59
Rubiaceae	*Gardenia leopoldiana* De Wild & T. Durand	—	Bark	4.89	0.65
	*Morelia Senegalensis* A. Rich. ex DC	—	Leaves	4.56	0.65
	*Stipularia africana* P. Beauv.	—	Leaves	4.00	0.63
Sterculiaceae	*Sterculia bequaertii* De Wild	Mvungela mfinda (Kikongo)	Seeds	3.44	0.54

**Table 2:** Ecological status of plants used in the management of sickle cell disease in Kikwit.

Plant species	HT	MT	BT	PD
*Justicia secunda* Vahl	Cult	Vh	Nph	Pan
*Rungia grandis* T. Ander	Fo	Vh	Ch	Pan
A*lternanthera bettzickiana* repens (L.) Link	Cult	Ah	Nph	Pan
*Annona senegalensis* Pers.	Sav	Sh	Mcph	AT
*Ceiba pentandra* (L.) Gaertn.	Cult ssp	T	Mgph	Pan
*Carica papaya* L.	Cult	T	Mcph	Pan
*Alchornea cordifolia* Müll. Arg.	Fo	Sh	Mcph	AT
*Bridelia ferruginea* Benth.	Sav	Sh	Mcph	GCZ
*Vigna unguiculata* (L.) Welp.	Cult	Ah	Th	AT
*Millettia laurentii* De Wild	Fo	T	Mgph	BGC
*Hypoxis angustifolia* Lam.	Cult	Ph	G	Pan
*Leocus africanus* (Bak. ex. Elliot) J. K. Morton	Sav,	Ssh	Mcph	GC
*Dissotis brazzae* Cogn.	Rud	Ssh	Th	Pan
*Albizia ferruginea* (Guill. et Piero) Benth.	Sav	T	Msph	GC
*Treculia africana* Decne	Fo	T	Msph	Pan
*Ficus asperifolia* Müg	Fo	Ssh	Lph	BGC
*Ficus tremula* Warb.	Fo	Sh	Mcph	GC
*Bougainvillea* sp comm. Ex Juss	Cult	Sh	Lph	Pan
*Hymenocardia acida* Tul	Sav	Sh	Mcph	AT
*Gardenia leopoldiana* De Wild & T. Durand	Fo	Sh	Mcph	AT
*Morelia senegalensis* A. Rich. Ex DC	Fo	Sh	Mcph	Pan
*Stipularia africana* P. Beauv.	Fal	Ssh	Mcph	Pan
*Sterculia bequaertii* De Wild	Fo	T	Mgph	GC

HT: habit types, MT: morphological types, BT: biotope types, PD: phytogeographical distribution, Cult: cultured, Cult ssp: subspontaneous cultured, Fo: forest, Fal: fallow, Rud: ruderal, T: tree, Sh: shrub, Ssh: subshrub, Ah: annual herb, Vh: vivace herb, Ph: perennial herb, PsPh: mesophanerophytes, McPh: microphanerophytes, NPh: nanophanerophytes, Ch: chamaephytes, Th: therophytes, G: geophytes, AT: Afro-tropical, BGC: Bas-Guinea-Congolese, GC: Guinea-Congolese, GCZ: Guinea-Congolese-Zambezean, and Pan: pantropical.

**Table 3:** *In vitro* antisickling activity of plant species used in the management of sickle cell disease in Kikwit.

N°	Plant species	Antisickling activity
1	*Albizia ferruginea* (Guill. et piero) Benth.	+
2	*Alchornea cordifolia* Müll. Arg.	+++
3	*Alternanthera bettzickiana* repens (L.) Link	+++
4	*Annona senegalensis* Pers.	+++
5	*Bougainvillea* sp comm. Ex Juss	+
6	*Bridelia ferruginea* Benth.	++
7	*Carica papaya* L.	++
8	*Ceiba pentandra* (L.) Gaertn.	+
9	*Dissotis brazzae* Cogn.	+++
10	*Ficus asperifolia* Müg	++
11	*Ficus tremula* Warb.	++
12	*Gardenia leopoldiana* De Wild & T. Durand	+
13	*Hymenocardia acida* Tul	++
14	*Hypoxis angustifolia* Lam.	+++
15	*Justicia secunda* Vahl	+++
16	*Leocus africanus* (Bak. ex. Elliot) J. K. Morton	++
17	*Millettia laurentii* De Wild	+
18	*Morelia senegalensis* A. Rich. ex DC	++
19	*Rungia grandis* T. Ander	++
20	*Sterculia bequaertii* De Wild	++
21	*Stipularia africana* P. Beauv.	++
22	*Treculia africana* Decne	++
23	*Vigna unguiculata* (L.) Welp	+++

+++: ≥80% of normalization rate; ++: 70% of normalization rate; +: 50% of normalization rate. An extract is considered to possess very high activity (+++) if normalization >70%; high activity (++) if 50< normalization <70%; weak activity (+) if 10< normalization <50%; no activity (−) if normalization <10% (14).

**Table 4:** Use values of medicinal plants used in the management of sickle cell disease in Kikwit city.

Plant species	Local *N*	Literature RFC	*N*	References
*Alchornea cordifolia* Müll. Arg.	0.69		0.130	[14, 23, 31]
*Annona senegalensis* Pers	0.71	2	0.087	[14, 18]
*Bougainvillea* sp comm. ex Juss	0.22	1	0.043	[31]
*Bridelia ferruginea* Benth.	0.32	2	0.087	[14, 31]
*Carica papaya* L.	0.79	3	0.130	[23, 31, 33]
*Ceiba pentandra* (L.) Gaertn.	0.24	2	0.087	[14, 31]
*Dissotis brazzae* Cogn	0.48	1	0.043	[9]
*Hymenocardia acida* Tul	0.81	2	0.087	[14, 31]
*Hypoxis angustifolia* Lam.	0.68	1	0.043	[31]
*Justicia secunda* Vahl	0.83	2	0.087	[23, 31]
*Treculia africana*	0.15	1	0.043	[31]
*Vigna unguiculata* (L.) Welp	0.81	3	0.130	[14, 31, 33]
Total sources	1	23	1	

**Table 5:** Perimeter, surface, and radius of untreated and treated sickle RBCs with the aqueous extract of *D. brazzae* and *A. bettzickiana* leaves.

Samples	Cellular perimeter (*μ*m)	Cellular surface (*μ*m^2^)	Cellular radius (*μ*m)
Negative control	24.9 ± 1.1	32.5 ± 2.6	—
Positive control	19.0 ± 1.7	25.1 ± 1.8	3.5 ± 0.3
Extract of *A. bettzickiana*	18.9 ± 1.7	24.7 ± 2.4	3.5 ± 0.4
Extract of *D. brazzae*	19.4 ± 2.1	25.3 ± 2.0	3.6 ± 0.5

## Data Availability

The data used in this study are provided and included within the article.
